# Integrating GPCR Regulation and Calcium Dynamics in Airway Smooth Muscle Function: A Comprehensive Review

**DOI:** 10.3390/cells15020203

**Published:** 2026-01-21

**Authors:** Saptarshi Roy, Vijaya Kumar Gangipangi, Pravesh Sharma, Rebecca E. Hancock, Pawan Sharma

**Affiliations:** 1 Department of Pathology and Laboratory Medicine, Lewis Katz School of Medicine, Temple University, Philadelphia, PA 19140, USA; 2 Thoracic Medicine and Surgery, Center for Inflammation and Lung Research, Lewis Katz School of Medicine, Temple University, Philadelphia, PA 19140, USA; 3 Aging + Cardiovascular Discovery Center, Lewis Katz School of Medicine, Temple University, Philadelphia, PA 19140, USA

**Keywords:** asthma, airway smooth muscle, airway remodeling, bronchoconstriction, calcium signaling, GPCR

## Abstract

Asthma is a heterogeneous disease that varies in clinical presentation, severity, and underlying biology but consistently involves airway remodeling (AR) and airway hyperresponsiveness (AHR), which is characterized by excessive airway narrowing in response to various stimuli. Airway smooth muscle (ASM) cells are primary contributors to airway hyperresponsiveness and bronchoconstriction. This review focuses on ASM cells and their role in asthma. We discuss the mechanisms by which ASM mediates AHR, increases airway thickness, and contributes to AR. Signaling through G protein-coupled receptors (GPCRs) regulates many ASM functions, including contraction, growth, and the synthetic activities that drive airway inflammation and remodeling. GPCR-dependent calcium flux serves as a key signaling axis controlling the contractile responses of ASM. Here we provide a comprehensive summary of the major GPCRs as well as other non-GPCRs identified in ASM cells. GPCR-induced calcium mobilization, downstream signaling and how it has been linked to specific ASM functions are also discussed. Furthermore, we highlight the clinical significance of targeting GPCRs in asthma therapy as well as recent development of novel therapeutics in the management of asthma. Thus, this review provides a comprehensive overview of airway smooth muscle in the context of asthma pathophysiology.

## 1. Introduction

Asthma is one of the most prevalent non-communicable diseases and is widely regarded as a heterogeneous condition characterized by chronic lung inflammation and airway remodeling, which together produce a broad range of clinical manifestations [1]. The 2018 Global Asthma Report states that asthma affects 339 million people worldwide, resulting in approximately 1000 deaths every day, and the prevalence of asthma varies significantly across different countries and regions [2,3]. In recent years, the trajectory of asthma has shifted from developed countries to developing countries due to rapid urbanization, increased use of fossil fuels and changing lifestyles [4]. Although the precise mechanisms triggering asthma remain unclear, both genetic and environmental factors play pivotal roles in disease onset. Due to its extensive heterogeneity, asthma is often used as an umbrella term encompassing multiple disorders that share common clinical features, including airway inflammation, bronchospasm, respiratory distress, and chronic cough [1]. This disorder arises from a complex interplay among immune cells including eosinophils, neutrophils, mast cells, macrophages, B and T lymphocytes and airway structural cells, such as epithelial cells and airway smooth muscle (ASM) cells, all of which contribute to its pathophysiological complexity [5,6,7]. Asthma that develops in early childhood is typically allergic in nature and associated with elevated serum IgE levels [8]. In contrast, adult-onset asthma does not necessarily require allergic sensitization and frequently exhibits reduced responsiveness to standard therapies, including inhaled corticosteroids [9,10]. Consequently, the traditional classification of asthma into allergic and non-allergic forms is increasingly recognized as an oversimplification, reflecting the broader challenge of defining and categorizing chronic inflammatory diseases.

Airway remodeling (AR) is a major pathological feature of asthma and contributes to long-term structural reorganization of molecular and cellular components within the airway wall. This process leads to airway narrowing, subepithelial fibrosis, increased extracellular matrix (ECM) deposition, accumulation of immune cells and fibroblasts, edema, angiogenesis, altered matrix composition, goblet cell metaplasia and excessive mucus production [11,12,13]. Although multiple cell types, including airway epithelial cells, ASM cells, fibroblasts, and immune cells, contribute to the development of AR in asthma and chronic obstructive pulmonary disease (COPD), ASM plays a particularly critical role in mediating airway constriction and remodeling. Airway hyperresponsiveness (AHR) is clinically defined as an exaggerated bronchoconstrictor response to stimuli that cause minimal or no airway narrowing in healthy individuals [13]. Moreover, an increase in ASM mass is considered one of the most critical factors contributing to elevated airway resistance, AR, and heightened responsiveness to contractile agonists [14,15]. G protein-coupled receptors (GPCRs) constitute the largest family of cell-surface receptors, and the majority of pro-contractile stimuli acting on ASM signal through GPCRs [16,17]. Activation of GPCRs initiates intracellular signaling via heterotrimeric G proteins, which regulate intracellular calcium ([Ca^2+^]_i_) concentration and calcium (Ca^2+^)-dependent signaling pathways, ultimately leading to ASM contraction [17]. In addition, mediators such as polypeptide growth factors and cytokines can modulate both Ca^2+^ flux and Ca^2+^ sensitivity in ASM, thereby influencing the magnitude and duration of GPCR-mediated responses [18]. In this review, we discuss the role of ASM in asthma, summarize the major GPCRs expressed on ASM and their downstream Ca^2+^-signaling mechanisms, and describe how these pathways contribute to ASM contraction and disease pathogenesis. Finally, we highlight current therapeutic strategies targeting these signaling networks, as well as potential drugs under development.

## 2. Airway Smooth Muscle Cells in Asthma

ASM cells are spindle-shaped, involuntary muscle cells located within the walls of the trachea and bronchioles, separated from the airway epithelium by the lamina propria [19]. They are essential structural components of the airway wall and mediate mechanical stretch as well as adaptive responses to environmental stimuli. Smooth muscle fibers are organized into bundles throughout the respiratory tract, forming a tissue network that attaches to bronchial cartilage in the large airways, while in smaller conducting airways, they are predominantly arranged in a spiral orientation relative to the airway axis [20]. Unlike striated muscle cells, which contain crystalline sarcomeres, ASM cells possess a single nucleus and lack a sarcomeric structure, enabling substantial phenotypic plasticity and continuous remodeling [21]. The presence of dense bodies enriched in α-smooth muscle actin (αSMA) is a defining feature of ASM cells; these structures serve as anchoring sites for actin filaments and play a critical role in ASM contraction [22].

### 2.1. Phenotypes

Primary cultures of ASM cells derived from enzymatically digested human lung tissue (trachea and bronchi) were first established in the 1980s and have since provided a reliable experimental system for characterizing ASM phenotypes [23]. Freshly isolated ASM cells typically retain a contractile phenotype, characterized by high expression of contractile proteins and robust responsiveness to contractile agonists. In contrast, exposure to serum-containing culture conditions induces a phenotypic shift toward a proliferative state, which is associated with reduced contractile responsiveness and downregulation of key contractile markers, including smooth muscle myosin heavy chain (smMHC), myosin light chain kinase (MLCK), and αSMA. This transition is accompanied by increased proliferative activity and enhanced synthesis of ECM components and cytokines [24,25]. Conversely, prolonged culture of ASM cells in serum-free conditions promotes expression of contractile markers, including αSMA, smMHC, desmin, calponin and M_3_ muscarinic receptors, resulting in a hypercontractile phenotype [26]. This dynamic process, termed phenotype plasticity, allows ASM cells to switch between contractile, synthetic, migratory, and proliferative states in response to inflammatory and mechanical cues [27]. While the contractile phenotype is most appropriate for studying ASM-mediated airway constriction, the proliferative phenotype is valuable for investigating the secretory and immunomodulatory functions of ASM cells. However, the in vivo relevance of this phenotype plasticity in asthma remains incompletely understood.

### 2.2. Airway Remodeling

**Figure 1:**
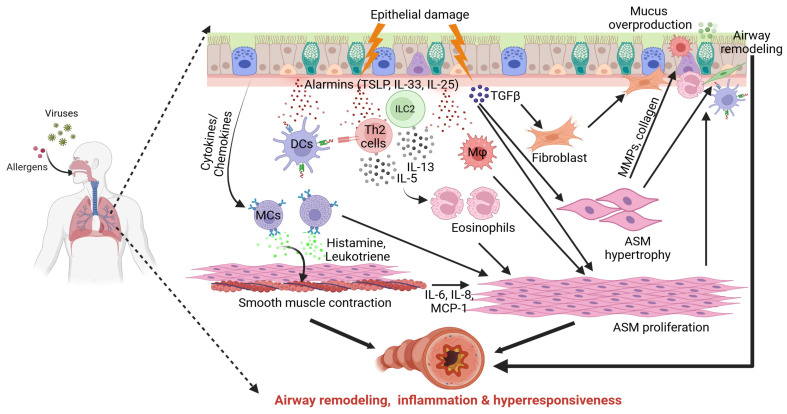
**Mechanism of airway remodeling, inflammation and hyperresponsiveness.** Asthma triggers by complex interactions between the airway epithelium with environmental allergens, respiratory viruses etc. Upon exposure to allergens, epithelial cells release soluble mediators such as alarmins (TSLP, IL-25, and IL-33) which recruit and activate immune cells such as ILC2, MΦ and MCs. Allergens can be captured by dendritic cells and presented to Th2 cells. The consequent activation of allergen-specific Th2 cells is responsible for the production of IL-5 and IL-13, which induce eosinophil maturation and survival. Epithelial cells and ASM cells secreted cytokines and chemokines which also cause MCs recruitment. Activated MCs release mediators such as histamine and leukotrienes which cause smooth muscle contraction. Epithelial tissue damage also releases TGF-β-mediated fibroblast proliferation and ASM hypertrophy. ASM releases MMPs and collagen. Inflammatory mediators and cytokines and chemokines cause ASM proliferation (hyperplasia). Secretion of collagen, mucus overproduction, ASM hypertrophy and hyperplasia and immune cells infiltration resulted in airway remodeling, inflammation and airway hyperresponsiveness. ASM: airway smooth muscle, DCs: dendritic cell, IL: interleukin, ILC2: innate lymphoid cell type 2, MΦ: macrophage, MCs: mast cells, MMPs: matrix metalloproteinases, TSLP: Thymic Stromal Lymphopoietin; Th2: T helper 2 cell, TGFβ: transforming growth factor beta.

AR is a multifaceted pathological hallmark of asthma, characterized by long-term alterations in airway architecture. These changes include ASM hyperplasia, subepithelial collagen deposition leading to thickening of the reticular basement membrane (RBM), disruption of epithelial barrier integrity accompanied by goblet cell metaplasia and consequent mucus hypersecretion, as well as enhanced angiogenesis. Collectively, these features are associated with most asthmatic phenotypes [28,29]. AR was first reported in 1922 in patients whose deaths were attributed to asthma, where necropsy examinations revealed extensive bronchial mucus plugging and marked thickening of the airway wall [30]. Notably, AR may be triggered either in the presence or absence of overt inflammation. During asthma, exaggerated mechanical forces caused by recurrent bronchoconstriction led to epithelial injury, thereby promoting AR through several mechanisms. These include transforming growth factor-β (TGF-β)-driven subepithelial fibrosis, fibroblast-to-myofibroblast transition, ASM hyperplasia, and mucus hypersecretion. Bronchoconstriction also induces the release of proinflammatory mediators such as IL-6, IL-8, and MCP-1, which act as mitogens for ASM proliferation (Figure 1) [31]. TGF-β and fibroblast growth factors further enhance ASM hypertrophy and ECM deposition, including collagens I and III and fibronectin, thereby altering the structural environment of the airway [32]. β_1_-integrins (e.g., α_2_β_1_, α_4_β_1_, and α_5_β_1_) regulate ASM growth and survival in response to ECM components, reinforcing remodeling processes. In parallel, ECM proteins such as fibronectin facilitate ASM migration and ECM turnover through matrix metalloproteinases (MMPs) [33,34,35]. These bidirectional interactions between ASM and ECM contribute to airway wall stiffening. In contrast, the “inflammation theory” proposes that airway epithelial cells (AECs) are primary drivers of AR [36]. Environmental allergens disrupt epithelial integrity and induce the release of alarmins, including IL-33, IL-25, and thymic stromal lymphopoietin (TSLP). These mediators activate innate and adaptive immune cells such as dendritic cells (DCs), type 2 innate lymphoid cells (ILC2), T helper 2 (Th2) lymphocytes, mast cells, and macrophages, thereby amplifying downstream inflammation. This process is accompanied by AECs’ apoptosis and paracrine secretion of TGF-β. IL-25 promotes fibroblast proliferation, while IL-33 induces fibronectin expression. Moreover, alarmins can directly stimulate lung fibroblasts to produce collagen, leading to ECM accumulation and subepithelial RBM thickening, an early event in asthma pathogenesis (Figure 1) [37]. Furthermore, increased ASM mass resulting from both hypertrophy (increase in cell size) and hyperplasia (increase in cell proliferation) plays a central role in AR and correlates strongly with disease severity and impaired lung function (Figure 1) [38,39]. Numerous studies have investigated the mechanisms underlying ASM hyperplasia. Growth factors such as TGF-β1, epidermal growth factor, and platelet-derived growth factor, as well as contractile stimuli acting through GPCRs, can induce hyperplasia in cultured ASM cells [40,41,42]. Additionally, ASM cells derived from asthmatic endobronchial biopsies proliferate more rapidly than those from healthy individuals, potentially due to reduced expression of the antiproliferative transcription factor C/EBPα, which also mediates corticosteroid-induced growth inhibition [43]. On the other hand, ASM hypertrophy occurs through at least two distinct pathways. One pathway involves activation of the mammalian target of rapamycin (mTOR), which induces phosphorylation of the 4E-binding protein (4E-BP), releasing the translation initiation factor eIF4E and promoting ASM cell hypertrophy [44]. In parallel, mTOR phosphorylates p70S6 kinase, leading to activation of S6 kinase and further contributing to ASM hypertrophy [45].

### 2.3. Airway Hyperresponsiveness (AHR)

AHR is defined as an exaggerated narrowing of the airways in response to chemical, physical, or pharmacological stimuli. ASM contraction can be induced by a wide range of agonists, including acetylcholine (ACh), histamine, endothelin-1, and bradykinin, which originate from neuronal terminals, adjacent airway cells, the circulation, or inhaled agents [20,46]. These stimuli may act directly on ASM through specific receptors, such as muscarinic, histaminergic, and leukotriene receptors, or indirectly via inflammatory mediators released in response to exercise, hypertonic aerosols, or adenosine [47]. ASM tone, defined as the baseline level of contraction that regulates airflow and airway resistance, is elevated in asthma and contributes significantly to increased AHR and contractility [48]. Enhanced ASM contractility is associated with disrupted Ca^2+^ homeostasis, increased Ca^2+^ sensitization, and altered airway innervation [49]. The nervous system plays a critical role in maintaining ASM tone, and dysregulation of neural control contributes to airway dysfunction in asthma. At the cellular level, ASM contraction is driven by interactions between myosin heads and actin filaments, generating force through cross-bridge cycling [50]. This force is transmitted via dense bodies, cytoskeletal filaments, adhesion junctions, and integrins that connect ASM cells to the ECM [51]. Dynamic cytoskeletal reorganization, including actin-myosin polymerization, further amplifies force generation during ASM activation [52]. Consequently, ASM force development reflects a balance between MLCK-mediated phosphorylation, MLCP-mediated dephosphorylation, and cytoskeletal remodeling processes [53]. Calcium sensitivity and MLCP inhibition are regulated primarily by RhoA/Rho-kinase signaling downstream of GPCRs. Activation of this pathway by multiple agonists enhances contractile force independently of [Ca^2+^]_i_, thereby contributing to sustained airway narrowing [54]. Clinically, the severity of AHR correlates strongly with asthma severity and is commonly assessed using methacholine or histamine challenge testing. Methacholine directly induces ASM contraction through muscarinic receptor activation, forming the basis for the methacholine challenge test. During this test, progressively increasing concentrations of methacholine are administered until a 20% reduction in forced expiration volume in one second (FEV_1_) is achieved. The concentration required to elicit this response, referred to as the provocative concentration (PC_20_), is typically <8 mg/mL in individuals with asthma, compared with >16 mg/mL in non-asthmatic subjects, reflecting heightened airway sensitivity [55].

### 2.4. ASM Beyond Contraction

ASM is now recognized as far more than a passive contractile element; it functions as an active immunomodulatory and structural regulator within the airway wall [56]. ASM cells possess intrinsic secretory capabilities and, under culture conditions, release a wide range of chemokines, cytokines, and growth factors in response to stimuli such as IL-1β, IL-13, and TNF-α, thereby contributing directly to airway inflammation and remodeling [57]. In addition, ASM exhibits proinflammatory and immunomodulatory properties through the secretion of mediators, including IL-5, IL-8, and IL-13, which promote cell proliferation and the recruitment of inflammatory cells such as mast cells and T lymphocytes (Figure 1) [58]. Brightling et al. and others have demonstrated that chemokines released by ASM, including CXCL10, CCL11, and CX3CL1, play a key role in mast cell recruitment to the ASM bundle [59,60]. Collectively, these mediators regulate the recruitment, activation, and survival of immune cells, positioning ASM as a central orchestrator of chronic inflammatory airway diseases such as asthma. Notably, ASM cells isolated from asthmatic individuals exhibit an enhanced capacity to release proinflammatory chemokines and ECM proteins, thereby amplifying airway inflammation and promoting increased cellular proliferation [61]. Beyond inflammation, ASM proliferation, migration, and phenotypic switching from a contractile to a synthetic state drive structural remodeling. These processes are characterized by increased ASM mass, excessive ECM deposition, and altered airway wall stiffness. Additionally, ASM expresses pattern-recognition receptors, including Toll-like receptors, enabling direct responses to pathogens and environmental signals. ASM also engages in bidirectional crosstalk with epithelial cells, fibroblasts, immune cells, and neuronal networks. Through these complex interactions, ASM can influence AHR independently of its classical contractile function [62]. Collectively, these findings underscore ASM as a multifunctional tissue capable of integrating mechanical, inflammatory, and remodeling signals that fundamentally shape the pathophysiology of airway disease.

## 3. Receptors Regulating Airway Smooth Muscle Function

GPCRs constitute the largest family of mammalian cell-surface receptors, accounting for more than 1% of the human genome [17]. GPCRs signal through direct interaction with heterotrimeric G proteins at the inner surface of the plasma membrane, where they function as nucleotide exchange factors that promote GDP release, enabling GTP binding and subsequent conformational activation of the G protein α subunit [63]. In ASM, contraction is primarily mediated by agonists that activate GPCRs coupled to the Gq family of heterotrimeric G proteins, with additional contributions from Gi-coupled receptors. GPCRs expressed on ASM cells play a central role in regulating airway resistance and structural remodeling [17]. In asthma, these receptors transduce extracellular signals from bronchoactive agonists such as ACh, histamine, and cysteinyl leukotrienes (Cys-LTs) into rapid and sustained increases in [Ca^2+^]_i_ concentration. Ca^2+^ acts as the primary molecular switch for ASM contraction and is a key contributor to AHR [17]. Beyond GPCR-mediated pathways, membrane-bound ion channels also play a critical role in modulating Ca^2+^ dynamics and contractile responses in ASM cells. Together, GPCRs and ion channels coordinate Ca^2+^ signaling to determine the magnitude and duration of ASM contraction.

Glucocorticoids remain the most effective therapeutic agents for asthma and exert their effects by binding to the glucocorticoid receptor, a ligand-activated nuclear receptor expressed in ASM cells. Activation of this receptor modulates gene transcription and contributes to bronchorelaxation, as well as suppression of inflammatory and remodeling pathways [64]. The key receptors expressed in ASM, their downstream signaling pathways, and their implications in asthma pathophysiology are summarized in Table 1.

### 3.1. Muscarinic Receptor

Muscarinic receptors are members of the GPCR family and play essential roles in regulating ASM tone, mucus secretion, and airway inflammation [65,66]. Among the five muscarinic receptor subtypes (M1–M5), the M2 and M3 receptors are the most relevant to airway physiology and pathophysiology [67]. These receptors are activated by ACh, released from postganglionic parasympathetic nerve terminals; however, they differ markedly in their anatomical distribution and functional contributions within the airway system [68].

M3 receptors are primarily expressed on the surface of ASM cells, where they mediate ASM contraction and mucus secretion [65,68]. They are also present in bronchial fibroblasts, AECs, and peripheral lung tissue [69]. Binding of Ach to the M3 receptor activates the Gq protein-coupled signaling pathway, stimulating phospholipase C (PLC), increasing intracellular inositol trisphosphate (IP_3_), and triggering Ca^2+^ release from the sarcoplasmic reticulum (SR) [70]. The resulting rise in intracellular Ca^2+^ induces ASM contraction, leading to bronchoconstriction [71,72]. Although this response contributes to normal airway defense and airflow regulation, excessive M3 receptor activation in asthma and COPD results in pathological airway narrowing and increased airway resistance [70].

Despite being more abundant than M3 receptors, M2 receptors regulate airway tone indirectly by inhibiting β-adrenergic signaling and suppressing adenylyl cyclase (AC) activity [66,73]. M2 receptors are located presynaptically on cholinergic nerve terminals, where they function as autoreceptors, and on postganglionic parasympathetic nerves with inhibitory roles [73,74]. Upon activation by Ach, M2 receptors further reduce Ach release from nerve terminals in the lung, trachea, and bronchi, thereby establishing a negative-feedback mechanism that limits excessive bronchoconstriction [69,73,75]. This feedback loop is critical for maintaining normal airway tone.

In airway inflammatory diseases such as asthma and COPD, elevated ACh release is associated with enhanced inflammation and AHR [66,71]. Eosinophils release major basic protein, which antagonizes M2 receptor function, disrupting the negative-feedback mechanism and increasing ACh availability. This imbalance promotes exaggerated M3 receptor-dependent bronchoconstriction [76]. Owing to their distinct roles, both M2 and M3 receptors represent important pharmacological targets in obstructive airway disease. Anticholinergic agents such as ipratropium and tiotropium act as muscarinic receptor antagonists that preferentially inhibit M3 receptors to prevent bronchoconstriction while preserving M2-mediated inhibitory feedback [69,73]. This selective blockade reduces ASM tone, improves airflow, and alleviates respiratory symptoms in patients with asthma and COPD. Table 2 summarizes the key cell lines and animal models used to study GPCR signaling in ASM, along with the major associated clinical findings.

### 3.2. Histamine Receptor

Histamine is a key mediator released from activated mast cells and contributes significantly to airway dysfunction in asthma through its interactions with multiple histamine receptor subtypes expressed either directly on ASM cells or indirectly within the airway wall. Among these, the histamine-1 receptor (H1R), which is abundantly expressed on ASM, plays a central role in allergic inflammation and is the primary mediator of histamine-induced bronchoconstriction [77,78]. In addition to ASM, H1R is expressed on airway nerves, respiratory epithelium, endothelial and hepatic cells, vascular smooth muscle, dendritic cells, and lymphocytes [79]. Activation of H1R by histamine stimulates Gαq/11-coupled signaling, leading to PLC activation, increased production of IP_3_, and elevation of [Ca^2+^]_i_ levels [80]. The resulting Ca^2+^ mobilization induces ASM contraction, airway narrowing, increased vascular permeability, and the production of prostacyclin and platelet-activating factor, collectively contributing to acute bronchospasm during asthma exacerbations [81]. In contrast, H2 receptors (H2R) couple to Gs proteins, stimulating adenylyl cyclase and increasing cyclic AMP (cAMP) levels, which promote ASM relaxation and attenuate contractile responses. However, in asthma, H2R-mediated bronchodilation is functionally outweighed by dominant H1-dependent constrictor signaling, particularly during allergen-induced mast cell degranulation when local histamine concentrations are markedly elevated [82].

Although H3R and H4R do not directly regulate ASM contraction, they influence airway physiology through neuromodulatory and immunomodulatory mechanisms. H3Rs are primarily localized to cholinergic and sensory nerve endings, where their activation inhibits neurotransmitter release, thereby modulating reflex bronchoconstriction and neurogenic inflammation [83,84]. In contrast, H4Rs are highly expressed on mast cells, eosinophils, and other immune cells, where they promote chemotaxis, cytokine release, and mastcells activation, amplifying airway inflammation and AHR [85]. Through these inflammatory and neural pathways, histamine indirectly enhances ASM sensitivity to contractile agonists, including histamine itself. Moreover, chronic airway inflammation and remodeling in asthma can alter histamine receptor expression and downstream signaling, further potentiating histamine-mediated bronchoconstriction and sustaining AHR.

### 3.3. Leukotriene Receptor

In allergic asthma, cytosolic phospholipase A2 (cPLA_2_) is activated in immune cells such as eosinophils, mast cells, and macrophage in response to elevated cytosolic Ca^2+^, levels, leading to the release of arachidonic acid from membrane phospholipids [86,87]. Arachidonic acid is subsequently metabolized by 5-lipoxygenase (5-LO) into 5-hydroperoxyeicosatetraenoic acid (5-HPETE), which is further converted into leukotriene (LT) A_4_, an unstable epoxide intermediate that serves as the precursor for LTB_4_ and the Cys-LTs, including LTC_4_, LTD_4_, and LTE_4_. These lipid mediators play a central role in driving inflammation and allergic responses in asthma [87]. LTB_4_ exerts its biological effects through specific GPCRs, namely BLT1 and BLT2 receptors (BLT1R and BLT2R). Both receptors contain a conserved DRY motif within their intracellular domains that is critical for interaction with the Gα_i_ subunit of the heterotrimeric G-protein complex [88,89]. Upon LTB_4_ binding, receptor activation induces GDP-to-GTP exchange on Gα_i_, resulting in dissociation of the Gα_i_ subunit from the βγ dimer. Activated Gα_i_ inhibits adenylyl cyclase (AC) and can engage downstream signaling pathways, including crosstalk with Gq-dependent mechanisms, ultimately promoting increases in [Ca^2+^]_i_ levels [90,91]. CysLTs primarily signal through their cognate GPCRs, CysLT_1_R and CysLT_2_R, which predominantly couple to Gq/11 proteins to activate PLC and elevate cytoplasmic Ca^2+^ concentrations. In addition, these receptors can engage Gi/o signaling pathways, leading to inhibition of AC activity and reduced cAMP production [92]. The resulting rise in [Ca^2+^]_i_ activates the contractile machinery of ASM, particularly MLCK, which phosphorylates myosin light chains to initiate ASM contraction. This process promotes airway narrowing and contributes to bronchoconstriction and the characteristic asthma symptoms of wheezing and dyspnea [93].

### 3.4. Prostaglandin Receptor

Prostaglandins are major lipid mediators involved in allergic airway inflammation and play a critical role in the pathogenesis of bronchial asthma. Approximately 10% of individuals with asthma experience acute bronchoconstriction triggered by aspirin or other NSAIDs that inhibit cyclooxygenase (COX) activity, a condition commonly referred to as aspirin-exacerbated respiratory disease [94]. Prostaglandin synthesis is catalyzed by COX enzymes, which convert arachidonic acid into prostaglandin derivatives that regulate inflammation, immune responses, and ASM tone [95]. COX enzymes initiate prostaglandin biosynthesis by incorporating two molecules of oxygen into arachidonic acid to form the unstable intermediate prostaglandin (PG)G_2_, which is subsequently reduced to PGH_2_. PGH_2_ serves as a common precursor for multiple biologically active prostanoids including PGE_2_, PGD_2_, PGF2α, prostacyclin (PGI_2_), and thromboxane A_2_ (TXA_2_). These metabolites are generated through cell- and tissue-specific synthases and isomerases, conferring distinct physiological and pathological effects within the airway [96]. PGE_2_ is the most widely distributed prostanoid and exerts its effects through four distinct GPCRs, EP_1_-EP_4_, each linked to unique intracellular signaling pathways [96]. Activation of EP_2_ and EP_4_ receptors stimulates AC, leading to increased cAMP production and resulting in predominantly anti-inflammatory and bronchodilatory effects. In contrast, EP_3_ receptor activation inhibits AC, reducing cAMP levels, while EP_1_ receptor signaling increases [Ca^2+^]_i_; both pathways are generally associated with leukocyte activation and proinflammatory responses [97]. Consistent with these mechanisms, PGE_2_ induces bronchodilation in both healthy individuals and patients with asthma or chronic bronchitis [98]. PGD_2_ mediates its biological effects through two GPCRs, DP1 and DP2. DP1 receptor activation couples to Gαs signaling, resulting in elevated intracellular cAMP levels, whereas DP2 receptor signaling occurs primarily via Gαi, leading to reduced cAMP and increased [Ca^2+^]_i_ [99,100]. Notably, DP2 shares structural similarity with chemoattractant receptors and plays a critical role in the recruitment and activation of inflammatory cells, including eosinophils and Th2 lymphocytes, thereby amplifying allergic airway inflammation. Another prostanoid, PGF2α, signals through the FP receptor, a Gq-coupled GPCR that elevates [Ca^2+^]_i_ concentrations and induces ASM contraction. In the respiratory tract, this signaling cascade promotes bronchoconstriction, an effect considered deleterious in the context of asthma pathophysiology [102,103].

### 3.5. Bitter Taste Receptor

Taste perception serves as a critical sensory defense mechanism in mammals by preventing the ingestion of potentially toxic substances. Bitter taste is detected by type 2 taste receptors (T2Rs), which are predominantly expressed on type II taste receptor cells within the taste buds of the tongue [104]. Upon activation, T2Rs trigger dissociation of the heterotrimeric G protein complex into Gα-gustducin and Gβγ subunits. The Gβγ subunit activates PLCβ2, leading to hydrolysis of PIP_2_ to generate IP_3_ and DAG. IP_3_ subsequently binds to IP_3_ receptors on the sarcoplasmic reticulum, releasing Ca^2+^ from intracellular stores [105]. Deshpande et al. were the first to demonstrate that T2Rs are expressed and functionally active in ASM cells [106]. Their study revealed that bitter tastants induce ASM relaxation through a T2R-dependent mechanism. Specifically, T2R produces localized increases in [Ca^2+^]_i,_ which in turn open Ca^2+^-activated K^+^ channels, leading to membrane hyperpolarization and subsequent ASM relaxation [106]. In isolated mouse airways, bitter tastants induced dose-dependent bronchodilation, and in a mouse model of asthma they exhibited bronchodilatory effects that were more potent than those of β_2_-agonists [106]. Importantly, T2R-mediated bronchodilation has also been demonstrated in human ASM cells derived from asthmatic and non-asthmatic donors [107]. T2R expressions and functions were preserved irrespective of disease status and were not impaired by IL-13-induced inflammatory conditions, suggesting that T2R signaling remains intact in asthmatic airways. Beyond their effects on ASM, T2Rs also play a role in immunomodulation [107]. Gene expression profiling of peripheral white blood cells from individuals with severe, therapy-resistant asthma and those with controlled asthma revealed marked upregulation of T2R transcripts [108]. Moreover, exposure of leukocytes from adult asthmatic patients to bitter agonists significantly suppressed LPS-induced cytokine release [108]. T2Rs have also been identified in human primary mast cells, where their activation by bitter agonists attenuated IgE-dependent secretion of pro-inflammatory mediators, including histamine and PGD_2_ [109]. Collectively, these findings indicate that T2Rs exert dual functions in the airway by promoting bronchodilation and suppressing inflammation. This unique combination of smooth muscle relaxation and immunoregulatory activity highlights T2Rs as promising therapeutic targets for the treatment of asthma.

### 3.6. Ion Channels

Ion channel receptors are critical modulators of asthma pathogenesis, influencing ASM tone, immune responses, and sensory nerve activation. By regulating ion flux across cellular membranes, these channels shape key processes underlying bronchoconstriction and AHR [110]. Among these receptors, transient receptor potential (TRP) channels constitute a diverse superfamily of cation channels expressed in AECs, ASM cells, and sensory neurons [112]. Several TRP channel subtypes, including TRPV1 (vanilloid), TRPA1 (ankyrin), and TRPM8 (melastatin), play essential roles in Ca^2+^ signaling and have been strongly implicated in asthma pathophysiology due to their involvement in airway inflammation, bronchoconstriction, and enhanced cough reflex sensitivity [111]. TRP channels respond to a wide range of physical and chemical stimuli, including allergens, environmental pollutants, temperature changes, and oxidative stress. Activation of these channels, particularly on airway sensory nerves, induces the release of neuropeptides such as substance P and calcitonin gene-related peptide (CGRP), thereby promoting neurogenic inflammation and contributing to AHR [112]. Notably, TRPA1 is highly sensitive to environmental irritants and industrial pollutants and is robustly activated under conditions of oxidative stress. Its activation provokes asthma-like symptoms, including wheezing, cough, dyspnea, and heightened airway sensitivity to diverse stimuli, highlighting its role as a key sensor linking environmental factors to airway dysfunction [121].

### 3.7. Nuclear Receptors

Studies have demonstrated that nuclear receptors (NRs) play significant roles in asthma pathogenesis, with certain NRs capable of either exacerbating disease or contributing to its control. These findings underscore the importance of NRs in maintaining immune homeostasis and regulating airway inflammation [121]. Among these receptors, the glucocorticoid receptor (GR) is central to asthma therapy and exerts potent anti-inflammatory and bronchoprotective effects, including modulation of ASM function through calcium-dependent signaling pathways. Upon ligand binding, GR translocates to the nucleus, where it regulates gene transcription to suppress inflammatory responses and reduces AHR. One important mechanism involves the regulation of Ca^2+^-activated potassium channels, particularly KCa3.1, in ASM cells. Inhibition of KCa3.1 has been shown to restore glucocorticoid sensitivity by preventing dephosphorylation of GRα at serine 211, a post-translational modification that impairs GR transcriptional activity and contributes to steroid resistance [122,123].

Peroxisome proliferator-activated receptor gamma (PPAR-γ) is another extensively studied NR in asthma because of its role in suppressing inflammation, regulating immune responses, and preventing airway remodeling [113]. The interaction between PPAR-γ signaling and calcium homeostasis is particularly relevant to asthma pathophysiology, influencing inflammatory cascades, ASM contractility, and structural remodeling. Activation of PPAR-γ reduces [Ca^2+^]_i_ levels in ASM and lung cells by downregulating voltage-gated Ca^2+^ channels and IP_3_ receptors. For instance, PPAR-γ agonists such as rosiglitazone suppress the expression of TRPC1 and TRPC6 channels in pulmonary arterial smooth muscle cells, thereby reducing store-operated Ca^2+^ entry (SOCE) and lowering cytosolic Ca^2+^ concentrations [114]. PPARγ ligands, including rosiglitazone and pioglitazone, have been shown to attenuate ASM proliferation, airway inflammation, and remodeling in asthma. Moreover, these agents can improve bronchodilator responsiveness and lung function, particularly in steroid-resistant asthma phenotypes, such as those observed in smokers [116].

Retinoid X Receptors (RXRs) represent another class of nuclear receptors that regulate gene expression in response to ligands such as retinoids and lipids. In the context of asthma, RXRs influence immune regulation, inflammatory signaling, and ASM behavior. While RXR activation may support certain protective and anti-inflammatory responses, inhibition of RXR signaling has also been proposed as a potential therapeutic strategy in settings where RXR activity contributes to airway inflammation or remodeling. However, the role of RXR modulation in asthma remains largely experimental, and further studies are required to define its therapeutic efficacy and safety profile [115].

## 4. Role of Calcium Signaling in Airway Smooth Muscle Contraction

GPCRs play a critical role in regulating both the contractile and proliferative phenotypes of ASM. In asthma, GPCRs transduce extracellular signals from bronchoactive agonists such as ACh, histamine, and Cys-LTs into rapid and sustained increases in [Ca^2+^]_i_. This Ca^2+^ signaling serves as the primary molecular switch for ASM contraction and contributes to AR and bronchoconstriction.

### 4.1. G Protein Coupling and Signal Transduction

**Figure 2:**
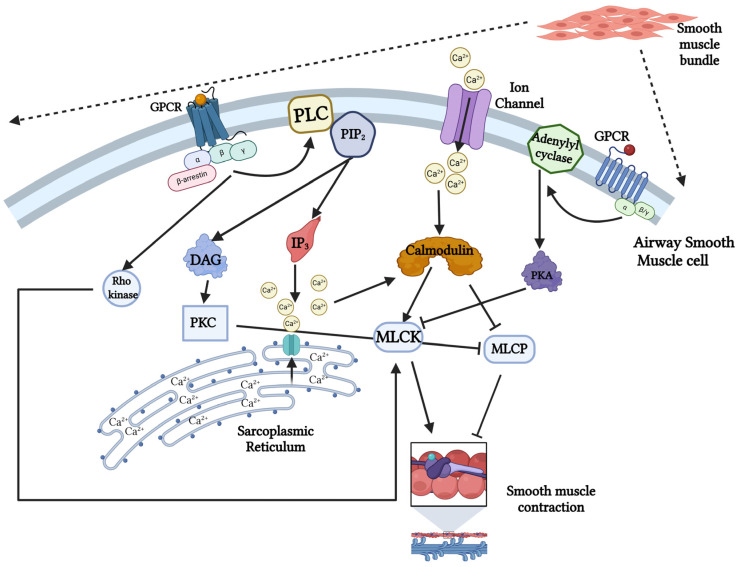
**Intracellular pathway for airway smooth muscle contraction.** Activation of GPCRs (such as muscarinic acetylcholine receptors) in ASM cells induced G protein-dependent activation of phospholipase C (PLC) which subsequently hydrolyzes 4,5-bisphosphate (PIP2) into inositol 1,4,5-trisphosphate (IP_3_) and diacylglycerol (DAG). IP_3_ induces the release of Ca^2+^ ions from the sarcoplasmic reticulum through an IP_3_-gated ion channel. Ion channel in the cell surface receptor also causes an uptake of extracellular Ca^2+^ ions from the extracellular environment. Intracellular Ca^2+^ ions, in turn, bind to calcium-sensing protein calmodulin (CaM) which activates myosin light chain kinase (MLCK), which phosphorylates and activates myosin to initiate cross-bridge formation and result in contraction. DAG, through protein kinase C (PKC) causes inhibition of myosin light chain phosphatase (MLCP) which promotes smooth muscle relaxation. The Rho-kinase pathway, which is stimulated by the activated GPCR, induces MLCK phosphorylation and contraction. Muscle relaxation is triggered by GPCRs such as β_2_-receptor stimulation, which activates adenylyl cyclase (AC) and increases cyclic AMP (cAMP) levels. The rise in cAMP activates PKA, which then carries out regulatory actions, including inhibition of MLCK.

Upon activation, GPCRs function as a guanine nucleotide exchange factor (GEF) for heterotrimeric G proteins, facilitating the exchange of GDP for GTP on the Gα subunit and thereby initiating downstream signaling cascades. Cellular responses are subsequently regulated by second messenger signaling pathways, which amplify and transmit signals from activated cell-surface receptors to intracellular effector systems. These pathways generate small, rapidly diffusing non-protein messengers that enable swift, coordinated, and highly regulated cellular responses. Two principals second- messenger systems downstream of GPCR activation are the cAMP pathway and the IP_3_/DAG pathway [124]. In the cAMP pathway, receptors coupled to Gs proteins activate AC, leading to the conversion of ATP into cAMP. cAMP primarily exerts its effects through activation of protein kinase A (PKA), which phosphorylates a wide range of target proteins, including enzymes, ion channels, and transcription factors, thereby regulating cellular function. In contrast, the IP_3_/DAG pathway is initiated by Gq-coupled receptors that activate PLC [125,126]. PLC hydrolyzes the membrane phospholipid PIP_2_ to generate two intracellular messengers: IP_3_ and DAG. IP_3_ diffuses through the cytosol to bind IP_3_ receptors on the SR, triggering Ca^2+^ release, whereas DAG remains associated with the plasma membrane to activate protein kinase C (PKC) [127]. The tightly synchronized generation and rapid degradation of these second messengers provide precise spatial and temporal control of GPCR signaling. This dynamic regulation allows cells to integrate diverse extracellular cues into amplified, cell-specific physiological responses as illustrated in Figure 2 [120,124,128].

### 4.2. Increase in Intracellular Calcium Level and Activation of Calmodulin

In eukaryotic cells, one of the most important and universal second-messenger signals is the transient increase in [Ca^2+^]_i_ concentration, which arises either from the release of Ca^2+^ from intracellular stores within the SR via IP_3_ receptors or through Ca^2+^ influx across the plasma membrane via calcium channels. To translate these rapid and dynamic changes in Ca^2+^ levels into specific biological responses, cells rely on the calcium-sensor protein calmodulin (CaM) [129]. CaM is a highly flexible, dumbbell-shaped protein containing four EF-hand motifs. Binding Ca^2+^ to these motifs occurs cooperatively and induces a pronounced allosteric conformational change from a closed to an open, activated state [130]. This structural rearrangement exposes hydrophobic interaction surfaces, enabling the Ca^2+^-CaM complex to associate with and regulate a wide range of target proteins. Among these targets is MLCK, a key effector that drives ASM contraction. CaM also activates integrator enzymes such as Ca^2+^/CaM-dependent protein kinase II, allowing it to function as a master decoder of Ca^2+^ signaling. Through these interactions, CaM interprets the frequency, amplitude, and duration of Ca^2+^ signals to regulate diverse cellular processes, including synaptic plasticity, ASM contraction, and transcriptional regulation, as illustrated in Figure 2 [129,131,132].

### 4.3. Myosin Phosphorylation, Dephosphorylation, and Contraction of Smooth Muscle

The contractile state of ASM is governed by a tightly regulated balance between two opposing GPCR-mediated second-messenger pathways. Bronchoconstriction is driven primarily by Gq-coupled receptors, such as the M3 muscarinic receptor, which activates PLC. PLC hydrolyzes PIP_2_ to generate IP_3_ and DAG. IP_3_ induces rapid mobilization of Ca^2+^ from the SR, and the resulting rise in [Ca^2+^]_i_ enables Ca^2+^-CaM-dependent activation of MLCK. MLCK phosphorylates myosin light chains, promoting actin-myosin cross-bridge cycling, ASM contraction and airway narrowing [133]. In contrast, bronchodilation is mediated by Gs-coupled receptors, most notably β_2_-adrenergic receptors, which stimulate AC to generate cAMP. Elevated cAMP activates PKA, which functions as a global inhibitory regulator of ASM contraction. PKA reduces [Ca^2+^]_i_ availability by inhibiting Ca^2+^ release and influx and directly phosphorylates and inactivate MLCK, thereby promoting ASM relaxation and airway dilation [17,120]. This signaling pathway represents the primary therapeutic target of rapid-acting and long-acting bronchodilator medications that are central to asthma management.

### 4.4. Subcellular and Microdomain Calcium Localization in ASM

Caveolae are specialized plasma membrane microdomains enriched in caveolin-1 that play a crucial role in shaping localized Ca^2+^ signals during inflammatory stimulation by spatially organizing key components of store-operated calcium entry (SOCE), including the Ca^2+^ channel Orai1 [134]. Although stromal interaction molecule-1 (STIM1) is primarily regulated within intracellular compartments and is less dependent on caveolar localization, pro-inflammatory cytokines such as TNF-α markedly enhance SOCE by upregulating Orai1 expression and promoting its recruitment to caveolar domains [135]. Importantly, even under inflammatory conditions, disruption of caveolar integrity significantly attenuates SOCE, highlighting the critical importance of Ca^2+^ microdomain organization in ASM Ca^2+^ dysregulation and inflammation-associated AHR [134,135].

In ASM cells, localized Ca^2+^ release events, commonly referred to as Ca^2+^ sparks and Ca^2+^ puffs, are primarily mediated by ryanodine receptors and IP_3_ receptors, respectively. These microdomain Ca^2+^ signals play a key role in regulating membrane excitability and contractile tension [136,137]. Ca^2+^ sparks can activate adjacent large-conductance Ca^2+^-activated K^+^ (BKCa) channels, leading to membrane hyperpolarization and functional relaxation of ASM [138]. Disruption of finely tuned microdomain interactions may impair BKca channel activation, resulting in enhanced contractility and reduced bronchodilatory capacity in diseased airways [139].

### 4.5. Spatiotemporal Microdomains and Paradoxical Relaxation

Recent advances have fundamentally reshaped our understanding of Ca^2+^ signaling by demonstrating that the spatiotemporal organization of Ca^2+^ signals rather than their absolute magnitude determines functional outcomes in ASM [140]. Distinct Ca^2+^ microdomains, oscillatory patterns, and signaling biases can paradoxically promote ASM relaxation even in the presence of elevated [Ca^2+^]_i_ [141,142]. A striking example of this phenomenon is *the T2R paradox*. T2Rs expressed on ASM cells induce potent bronchodilation despite increasing Ca^2+^ levels [106,141]. High-resolution spatiotemporal Ca^2+^ mapping has revealed that, whereas histamine evokes synchronized, global Ca^2+^ waves that drive contraction, TAS2R agonists such as chloroquine generate asynchronous, spatially restricted peripheral Ca^2+^ signals characterized by higher entropy and limited propagation [106,141]. These localized Ca^2+^ transients are confined to membrane-adjacent microdomains, where they selectively activate BKCa channels. The resulting K^+^ efflux hyperpolarizes the plasma membrane, leading to closure of voltage-gated Ca^2+^ channels and promoting robust ASM relaxation, culminating in marked bronchodilation [141]. Beyond T2Rs, biased agonism further illustrates how GPCR signaling can diverge from classical Ca^2+^-dependent contraction. The M3 muscarinic receptor, traditionally viewed as exclusively pro-contractile via Gq-mediated PLC activation, can signal through alternative pathways depending on ligand bias. The biased ligand PD102807 selectively engages β-arrestin-dependent signaling without triggering Gq-mediated Ca^2+^ influx. This pathway activates AMP-activated protein kinase (AMPK), which suppresses mTORC1 signaling and inhibits the hypercontractile and pro-remodeling phenotype characteristic of chronic asthma [143,144]. The findings challenge the canonical view of muscarinic signaling and highlight receptor bias as a mechanism for decoupling Ca^2+^ elevation from contraction. Muscarinic GPCRs also influence Ca^2+^ dynamics through non-canonical lipid signaling pathways. In addition to PLC-mediated signaling, muscarinic receptor activation engages the sphingosine kinase (SPHK)/sphingosine-1-phosphate (S1P) axis. In peripheral airways, pharmacological inhibition of SPHK significantly attenuates muscarinic agonist-induced constriction, indicating that SPHK/S1P signaling amplifies GPCR-driven [Ca^2+^]^i^ increase. Notably, genetic depletion of muscarinic receptors does not alter SPHK expression, suggesting functional coupling rather than transcriptional regulation. This pathway represents an additional lipid-mediated mechanism contributing to Ca^2+^-dependent ASM contraction and cholinergic hyperresponsiveness in airway diseases [145]. Mechanosensitive pathways further expand the concept of Ca^2+^-dependent relaxation. The mechanically activated ion channel Piezo1 functions as a non-canonical regulator of ASM tone. Chemical activation of Piezo1 with Yoda1 induces localized Ca^2+^ influx that, similar to T2R signaling, preferentially activates BKCa channels and promotes ASM relaxation. Importantly, this mechanism bypasses the receptor desensitization commonly associated with β2-adrenoceptors agonists, positioning Piezo1 as an alternative bronchodilatory pathway [143,146]. Finally, calcium-sensing receptors (CaSR) represent a therapeutically actionable GPCR pathway that directly links Ca^2+^ dysregulation to AHR and inflammation. CaSR expression is upregulated in asthmatic ASM, and receptor activation enhances Ca^2+^ signaling, AHR, and inflammatory responses in both human ASM cells and animal models. Conversely, pharmacological inhibition or genetic deletion of CaSR in ASM attenuates Ca^2+^ signaling and reduces bronchial hyperreactivity, underscoring its pathogenic role in asthma [147,148]. Collectively, these findings reinforce that ASM function is governed not simply by Ca^2+^ abundance, but by the spatial confinement, temporal dynamics, and signaling context of Ca^2+^ signals. Spatiotemporal Ca^2+^ microdomains, biased GPCR signaling, mechanosensitive channels, and non-canonical lipid pathways together define whether Ca^2+^ drives contraction, relaxation, or remodeling, offering novel conceptual and therapeutic avenues for targeting AHR in asthma.

## 5. Therapeutic Approach Targeting GPCR Signaling

GPCRs constitutes the largest family of drug targets in humans, and GPCRs expressed on ASM cells are the direct targets of most currently available anti-asthma therapies [17]. Although inhaled corticosteroids (ICSs) remain the cornerstone of asthma management and are effective for the majority of patients, approximately 5–10% of individuals remain uncontrolled despite high-dose ICSs and systemic corticosteroid therapy [149]. Advances in the understanding of asthma pathophysiology and ASM biology have driven a paradigm shift in the management of severe asthma from nonspecific bronchodilators and corticosteroids toward targeted biologic therapies, with the promise of increasingly precise and disease-modifying treatments.

### 5.1. Muscarinic and Leukotriene Receptor Antagonists

Muscarinic receptor antagonists were among the earliest GPCR-targeted therapies introduced for obstructive airways disease. Ipratropium bromide was first introduced in the 1970s, followed by oxitropium bromide and the longer-acting tiotropium bromide, which provided sustained bronchodilation [150]. Inhaled long-acting muscarinic antagonists (LAMAs), including glycopyrronium bromide and umeclidinium bromide, were initially approved for COPD in 2010. The addition of a LAMA to ICS or long-acting β_2_-agonist (LABA) therapy is now an established and validated strategy for the treatment of severe asthma [150,151]. These anticholinergic agents provide symptomatic relief by reducing bronchoconstriction and mucus secretion through antagonism of M1 and M3 muscarinic receptors [152]. Leukotriene receptor antagonists (LTRAs), including pranlukast, zafirlukast, and montelukast, were introduced in the late 1990s. These agents exert anti-inflammatory and modest bronchodilatory effects by blocking CysLTs at the CysLT_1_R, thereby attenuating leukotriene-driven airway inflammation and bronchoconstriction.

### 5.2. Adrenergic Agonists

Epinephrine is a well-established short-acting, nonselective adrenergic agonist that induces ASM relaxation primarily through stimulation of β_2_-adrenergic receptors. The introduction of inhaled β-agonists in the 1960s revolutionized asthma therapy by enabling rapid symptom relief and establishing β_2_-agonists as a central component of asthma management [153]. Salbutamol became the most widely used β-adrenergic agonist and laid the foundation for the development of additional short-acting β_2_-agonists (SABAs), including carbuterol, clenbuterol, and fenoterol [154]. Long-acting β_2_-agonists (LABAs), such as salmeterol were introduced in the 1980s and provided bronchodilation lasting up to 12 h. More recently, ultra-long-acting β_2_-agonists (ultra-LABAs) have been developed to sustain bronchodilation for up to 24 h, enabling once-daily dosing and improved treatment adherence [151].

### 5.3. Corticosteroids

Since their first clinical use in 1956 for the treatment of acute asthma, corticosteroids have remained central to asthma therapy [155]. Subsequent agents, including prednisone and dexamethasone, reduced mineralocorticoid activity, while the introduction of inhaled beclomethasone in 1972 enabled effective disease control with fewer systemic adverse effects. Later studies confirmed the efficacy of oral, intravenous, and inhaled corticosteroids, including triamcinolone, budesonide, and fluticasone [151]. Glucocorticoids are the primary corticosteroids used in contemporary asthma management due to their potent anti-inflammatory effects and ability to reduce AHR. These agents bind cytosolic glucocorticoid receptors, which translocate to the nucleus and regulate gene transcription by inducing anti-inflammatory genes while repressing proinflammatory cytokines, chemokines, adhesion molecules, and key inflammatory enzymes [156].

### 5.4. Mast Cell Stabilizers

Mast cells play a central role in asthma pathogenesis through the release of vasoactive and proinflammatory mediators, promising early therapeutic efforts to target mast cell activation [157]. Cromones, including cromolyn sodium and nedocromil, were classified as “mast cell stabilizers” because of their ability to inhibit mediator release following IgE cross-linking. However, sodium cromoglycate was found to reduce mediator release only modestly (approximately 10–20%) and to induce rapid tachyphylaxis in vitro, limiting its long-term clinical utility [158].

### 5.5. Biologic Therapies and Emerging Interventions

Asthma management underwent a major transformation in the early 2000s with the approval of omalizumab, the first biologic therapy for asthma in the United States and Europe [159]. Omalizumab is a recombinant humanized monoclonal antibody targeting IgE, a key driver of the allergic inflammatory cascade implicated in approximately 70% of asthma cases [160]. Subsequently developed biologics have expanded treatment options for severe asthma. Dupilumab, an anti-IL-4/IL-13 monoclonal antibody, has demonstrated the ability to attenuate ASM remodeling and contractility and showed strong efficacy in phase 3 clinical trials [161,162]. Additional USFDA-approved biologics include tezepelumab, which targets TSLP, and the anti-IL-5 mepolizumab and reslizumab [163,164,165]. Despite their clinical efficacy, the direct effects of these biologics on ASM behavior and AHR remain incompletely understood. Bronchial thermoplasty represents an alternative treatment for adults with severe refractory asthma. This minimally invasive procedure delivers controlled thermal energy to reduce ASM mass [166]. Clinical follow-up studies have demonstrated improvements in symptom-free days, reduced exacerbations, and enhanced expiratory airflow and airway responsiveness [167]. More recently, the oral selective DP_2_ antagonist fevipiprant has shown promise in targeting ASM remodeling. In a phase II clinical trial involving bronchial biopsies, fevipiprant significantly reduced ASM mass, likely by limiting recruitment of myofibroblasts and fibrocytes into ASM bundles, in addition to decreasing eosinophilic inflammation [119]. A deeper understanding of ASM mechanics and immunomodulatory functions is expected to facilitate the development of future therapies that directly target disease-driving mechanisms.

## 6. Conclusions

In summary, ASM plays a central and multifaceted role in the pathophysiology of asthma, acting not only as the primary effector of bronchoconstriction but also as an active immunomodulatory and structural component of the airway wall. Dysregulated Ca^2+^ signaling, altered GPCR-mediated contractile and relaxation pathways, and inflammatory mediator-driven remodeling collectively contribute to AHR and increased ASM mass, thereby reinforcing disease progression. Despite significant advances in pharmacological treatment, most current treatments primarily target symptomatic relief rather than addressing the underlying mechanisms of ASM dysfunction, and their effectiveness varies across heterogeneous asthma phenotypes. Continued investigation into the molecular, biomechanical, and immunological regulation of ASM, including contractile signaling, remodeling pathways, receptor biology, and ion channel modulation, will be essential for the development of more precise, phenotype-specific, and disease-modifying therapies. A comprehensive understanding of ASM behavior, therefore, represents a critical frontier in transforming asthma management and improving long-term patient outcomes.

## Figures and Tables

**Table 1:** Summary of GPCRs, G proteins, and key signaling pathways.

Receptor Type	Examples/Ligand	Signaling Mechanism	Key Second Messengers/Effectors	Role in Airway Hyperresponsiveness (AHR)	References
Muscarinic receptors	M1, M2, M3; ligand: Acetylcholine	Gq (M1, M3) → PLC → IP3/DAG → Ca^2+^/PKC; Gi (M2) → inhibits AC	↑ Ca^2+^, ↑ PKC, ↓ cAMP (M2)	M3 mediates bronchoconstriction, mucus secretion; contributes to AHR	[65,66,67,68,69,70,71,72,73,74,75,76]
Histamine receptors	H1, H2, H3, H4; ligand: Histamine	H1: Gq → PLC → IP3/DAG → Ca^2+^/PKC H2: Gs → AC → cAMP → PKA	↑ Ca^2+^ (H1), ↑ cAMP (H2)	H1: bronchoconstriction, vascular permeability; H2: bronchodilation	[77,78,79,80,81,82,83,84,85]
Leukotriene receptors	CysLT_1_, CysLT_2_; ligand: LTC_4_, LTD_4_, LTE_4_	Gq → PLC → IP3/DAG → Ca^2+^/PKC	↑ Ca^2+^, PKC activation	Airway smooth muscle contraction, inflammatory cell recruitment → AHR	[86,87,88,89,90,91,92,93]
Prostaglandin receptors	EP1-4, DP1-2, FP, TP, IP; ligand: PGE2, PGD2, PGF2α, TXA2, PGI2	EP1, FP, TP: Gq → PLC → IP3/DAG → Ca^2+^/PKC EP2, EP4, IP: Gs → AC → cAMP → PKA	↑ Ca^2+^ or ↑ cAMP depending on subtype	EP1/FP/TP: bronchoconstriction; EP2/EP4: bronchodilation	[94,95,96,97,98,99,100,101,102,103]
Bitter taste receptors (T2Rs)	T2R family; ligand: bitter compounds	Gi → inhibits AC → ↓ cAMP; some Gβγ → PLC → IP3/DAG → ↑ Ca^2+^	↓ cAMP, ↑ Ca^2+^ locally in cilia	Bronchodilation via local Ca^2+^-dependent NO release; protective against AHR	[104,105,106,107,108,109]
Ion channels	TRPV1, CFTR, BK channels	Direct ion flux (Ca^2+^, Cl^−^, K^+^)	↑ Ca^2+^ (TRPV1), Cl^−^ transport (CFTR), K^+^ efflux (BK)	TRPV1: sensory nerve activation → bronchoconstriction, cough; CFTR/BK: regulate airway tone	[110,111,112]
Nuclear receptors	Glucocorticoid receptor, Vitamin D receptor, PPARγ	Ligand diffuses → receptor-ligand complex binds DNA → transcription	Gene expression changes	Anti-inflammatory effects, reduce AHR	[113,114,115,116]

**Table 2:** Summary of Key Cell Lines, Animal Model, and Clinical Findings on GPCR Signaling in Airway Smooth Muscle.

Model	GPCR/Pathway	ASM-Related Finding	Asthma Relevance	References
Cell culture (human ASM)	M3 (Gq–PLC–IP_3_–Ca^2+^)	↑ Ca^2+^, MLCK activation → contraction & proliferation	Pro-contractile signaling in AHR	[65,66,67,68,69,70,71,72]
	Histamine H1 (Gq)	Ca^2+^-mediated contraction	Bronchospasm in allergic asthma	[77,78,79,80,81]
	CysLT_1_ (Gq/Gi)	Ca^2+^ rise → contraction & inflammatory mediator effects	Leukotriene-driven AHR	[86,87,88,89,90,91,92,93]
	EP2/EP4 (Gs)	↑ cAMP → ASM relaxation & anti-inflammatory effects	Endogenous bronchodilation	[96,97,98]
	Bitter taste receptors (TAS2Rs)	Distinct Ca^2+^ signaling + RhoA/MYPT1 regulation → relaxation despite Ca^2+^ rise	Novel bronchodilation independent of β-agonists; potential target	[117]
	G_12_/_13_-linked signaling	RhoA/ROCK-mediated Ca^2+^ sensitization and contraction	Alternative pro-contractile pathway beyond classical Gq	[118]
Animal models	M3, CysLT_1_	Enhanced airway resistance, AHR	Validates contractile GPCR roles in vivo	[70,72,93]
	DP2 (CRTH2)	Promotes eosinophilia & Th2 inflammation	Pathogenic inflammation linked to remodeling	[99,100,101]
	TAS2R activation	Bronchodilation & inhibition of Ca^2+^-induced contraction	Emerging therapeutic mechanism in vivo	[106]
Clinical/Translational	Antimuscarinics (M3 blockers)	Improved airflow, reduced bronchoconstriction	Established therapeutic target	[69,73]
	Leukotriene antagonists	Reduced AHR & symptoms	Confirms leukotriene role	[93]
	DP2 antagonists	↓ ASM mass, ↓ eosinophilia	Potential remodeling modulation	[101,119]
	β_2_-agonists	cAMP-mediated bronchodilation	Standard therapy targeting Gs signaling	[17,120]
	Novel bitter TAS2R agonists	Potent bronchodilation with distinct pathway	Potential new class of bronchodilators	[106]

## Data Availability

No new data were created or analyzed in this study.

## Abbreviations

The following abbreviations are used in this manuscript:

AC	adenylyl cyclase;
Ach	acetylcholine;
AECs	airway epithelial cells;
ASM	airway smooth muscle;
AR	airway remodeling;
AHR	airway hyperresponsiveness;
αSMA	α-smooth muscle actin;
BLTR	BLT receptor;
cAMP	cyclic AMP;
COPD	chronic obstructive pulmonary disease;
Ca^2+^	calcium;
[Ca^2+^]_i_	intracellular calcium;
CaM	calmodulin;
COX	cyclooxygenase;
Cys-LTs	Cysteinyl Leukotrienes;
DAG	diacylglycerol;
ECM	extracellular matrix;
GPCR	G protein-coupled receptor;
GR	glucocorticoid receptor;
H1R	Histamine-1 receptor;
IP3	inositol trisphosphate;
LAMAs	long-acting muscarinic antagonists;
LABA	long-acting β_2_-agonist;
MLCK	myosin light chain kinase;
MLCP	myosin light chain phosphatase;
MMPs	matrix metalloproteinases;
NRs	nuclear receptors;
PLC	phospholipase C;
PPAR-γ	proliferator-activated receptor gamma;
PKC	protein kinase C;
PKA	protein kinase A;
RBM	reticular basement membrane;
SR	sarcoplasmic reticulum;
smMHC	smooth muscle myosin heavy chain;
SABAs	short-acting β-2 agonists;
SOCE	store-operated calcium entry;
T2Rs	type 2 taste receptors;
ILC2	type 2 innate lymphoid cells;
TGF-β	transforming growth factor-beta;
TRP	Transient Receptor Potential
